# Adverse Childhood Experiences and Mindfulness in Chinese College Students During the COVID-19 Pandemic

**DOI:** 10.3389/fpsyt.2021.619128

**Published:** 2021-05-26

**Authors:** Chienchung Huang, Meifen Yang, Yun Geng, Yafan Chen, Shannon P. Cheung, Guosheng Deng, Qiang Dong, Hongwei Hu, Kai Hua, Jinyu Liao, Yuanfa Tan, Bin Tu, Enjian Wang, Zhihong Yu, Congcong Zhang, Shuyan Zhang, Gaosheng Zhuo

**Affiliations:** ^1^School of Social Work, Rutgers, The State University of New Jersey, New Brunswick, NJ, United States; ^2^School of Public Administration, Guangdong University of Foreign Studies, Guangzhou, China; ^3^School of Government, Central University of Finance and Economics, Beijing, China; ^4^School of Public Policy & Management, Tsinghua University, Beijing, China; ^5^College of Humanities and Development Studies, China Agricultural University, Beijing, China; ^6^School of Public Administration and Policy, Renmin University of China, Beijing, China; ^7^Soccer Academy, Wuhan Sports University, Wuhan, China; ^8^Research Institute of Social Development, Southwestern University of Finance and Economics, Chengdu, China; ^9^School of Humanities and Social Sciences, North China Electric Power University, Beijing, China; ^10^School of Sociology, Wuhan University, Wuhan, China; ^11^Department of Youth Work Research, China Youth University of Political Studies, Beijing, China; ^12^School of International and Public Affairs, Jilin University, Changchun, China; ^13^Institute of Social Development, Wenzhou University, Wenzhou, China

**Keywords:** mindfulness, China, adverse childhood experiences, college, pandemic

## Abstract

Mindfulness has been found to have many positive effects on life outcomes, including mental health and educational achievement. However, less is known about the antecedents of mindfulness, particularly in Chinese college students. This study examines the effect of adverse childhood experiences (ACEs) on mindfulness among Chinese college students in September 2020, during the COVID-19 pandemic. We hypothesized that ACEs negatively affected students' mindfulness. The data were collected from 1,871 college students from 12 colleges across China. The results aligned with our hypothesis that ACEs was negatively associated with mindfulness. In particular, emotional abuse and neglect in childhood appear to have the most negative effects on mindfulness compared to other dimensions of ACEs such as physical abuse and household challenges.

## Introduction

Mindfulness is a state of consciousness that incorporates purposeful awareness and attention, as well as non-judgmental reactions, to the present moment (1). It has two key components, mindful attention and mindful metacognition (2–4). Mindfulness starts with bringing awareness to the present moment by regulating attention, which makes individuals alert to what is occurring in the here-and-now. Mindful metacognition refers to detachment from the monitoring of thoughts and feelings about the ongoing events. Such detachment requires individuals to step back mentally, rather than getting involved and judging their thoughts and feelings. These components, mindful attention and mindful metacognition, have been shown to be highly correlated with one another (*r* = 0.44, *p* < 0.001) (4). Empirical studies have found that mindfulness increases students' social and emotional competence (5, 6) and academic performance (7, 8), while reducing behavioral problems (9, 10). Although extensive evidence has shown that mindfulness has positive effects on life outcomes, the antecedents of mindfulness are relatively understudied (4, 8). Given that mindfulness is associated with positive outcomes, it is imperative to identify its antecedents so that we can detect high-risk groups with low levels of mindfulness and provide necessary support and services to them.

Mindfulness relies on a capacity to self-regulate attention and the interplay of three motivational forces that drive cognitive resources into or away from self-regulation. The three motivational forces include “metacognitive beliefs that drive resources into self-regulation, mental fatigue that draws resources away from self-regulation, and situational appraisals that influence how much self-regulation is needed to maintain mindfulness” [(4), p. 79]. Using three different samples of college students, hospital nurses, and full-time workers in the community, Reina and Kudesia (4) found that participants' capacity to self-regulate and their metacognitive beliefs were positively related to mindfulness. By contrast, mental fatigue and situational stressors were negatively associated with mindfulness.

Adverse childhood experiences (ACEs) are negative events that occur before a person reaches 18 years of age. These events may include abuse (i.e., psychological, physical, or sexual), neglect, household challenges (i.e., violence perpetrated against mother; cohabitation with individuals who use substances, have mental illness, or have been incarcerated) (11). These events typically have harmful after-effects and may even be conceptualized as traumatic (11). ACEs have been shown to be associated with poorer functioning related to mental health, somatic disturbances, substance abuse, impaired memory, early sexuality, perceived stress, anger control, and risk of intimate partner violence (12–14). For example, Anda et al. (12) conducted a study on 17,337 adults and found that higher ACE score significantly increased the risks of the affective, somatic, substance abuse, memory, sexual, and aggression-related outcomes. Moreover, the mean number of comorbid outcomes tripled across the range of ACE scores (12). Likewise, Merrick et al. (13) investigated ACEs in 25 states between 2015 and 2017 and found that about one in six adults experienced four or more types of ACEs. ACEs were significantly associated with worse health outcomes, greater health risk behaviors, and greater socioeconomic challenges. Thus, it is likely that ACEs reduce self-regulation capacity and metacognitive beliefs while increasing the likelihood of mental fatigue, all of which lead to reductions in degree of mindfulness. Given that ACEs have significant detrimental effects on individual development, it is critical to investigate the extent to which ACEs may affect mindfulness.

The conceptual framework of this study utilizes Herman's (15) trauma theory, which posits that traumatic experiences, including those events considered ACEs, can cause considerable harm to individual well-being. The effects of trauma impose negative consequences to both “psychological structures of the self” and “the systems of attachment and meaning that link individual and community” [(15), Chapter 3, para. 2]. Underlying the presentation of trauma symptom clusters are the traumatized individuals' fractured belief systems surrounding trust and safety in the world (15). These three symptom clusters, which are often characteristic of clinical posttraumatic stress disorder (PTSD), are termed hyperarousal, constriction, and intrusion.

A key symptom of PTSD, hyperarousal is the overactivation of the sympathetic nervous system in response to a traumatic memory. Individuals experiencing chronic hyperarousal may live in an extended state of self-protective vigilance. Affected individuals may find it difficult to regulate this hyperarousal. Often, to counter this, individuals may experience numbing, or constriction. This leads individuals to remain physiologically, emotionally, and cognitively unresponsive to stimuli. Although constriction may be conceptualized as having a protective function by helping individuals avoid trauma responses, the experience of intrusion symptoms causes a sudden and intense reliving of the traumatic event: disjointed images and graphic sensations of the original experience, typically in the form of nightmares, can disrupt constriction.

Traumatic events may overwhelm individuals by causing severe disruptions to their core beliefs regarding safety and trust. This subsequently leaves them feeling powerless and disconnected; some even feel as though they have lost all meaning in life. In response to extreme trauma, individuals become hypersensitive to potential danger and/or dissociative, reducing their capacity to maintain a state of mindfulness (15, 16). In fact, previous mindfulness literature has identified that the areas of the brain associated with mindfulness are responsible for basic functions such as body regulation, conditioned fear modulation, and emotional balancing, among others (17). These few functions, however, are heavily dysregulated in individuals who exhibit posttraumatic stress symptoms. Regulating the conditioned fear response, what Forner (17) refers to as “updating our files,” allows individuals to differentiate and distinguish a stimulus that was once a valid fear but is now no longer a threat. For example, two feet of water may appear scary and life-threatening to a toddler but less so to an adult (17). Hypervigilance indicates this inability to regulate the conditioned fear response, leaving an individual in a state of heightened alertness and unable to remain non-judgmental (15). On the other side of the spectrum of trauma responses, constriction or numbing similarly indicates a “shutting down” of the body, whereby an individual may be taken out of the present moment to avoid potentially triggering stimuli and unable to engage in mindfulness (16). Whether an individual tends to experience hypervigilance, constriction, or a combination of both, these symptom clusters appear to affect the same functions that are performed by the areas of the brain associated with mindfulness, indicating a potential negative relation between trauma and mindfulness.

Empirical evidence indicates that ACEs are negatively associated with mindfulness; this is true across applications of various mindfulness measurement instruments (16, 18, 19). Emirtekin et al. (18) found that adolescents' dispositional mindfulness—defined as open and receptive awareness of and attention to the present moment and measured by the Mindful Attention Awareness Scale (MAAS)—has a significant negative correlation with childhood emotional abuse (*N* = 470; *r* = −0.46; *p* < 0.001). In another study by Nagel et al. (19), there was a significant negative association (*p* < 0.001) between ACEs and dispositional mindfulness, measured by the Revised Cognitive and Affective Mindfulness Scale (CAMS-R). The CAMS-R measures dispositional mindfulness on a scale of 12 to 48. In Nagel's et al. (19) study of over 700 adult patients, those without ACEs had an average CAMS-R score of 35.2, while those with 3 or more ACEs had a significantly lower average score of 32.5. Last, using the Revised Mindfulness Self-Efficacy Scale (MSES-R) to measure facets such as emotion regulation, equanimity, and distress tolerance, Voith et al. (16) reported that ACEs were negatively correlated with mindfulness self-efficacy (*r* = −0.41) in a sample of approximately 70 men of color.

While the extant literature has provided substantial evidence of the relation between ACEs and mindfulness, most studies are situated in the context of Western countries. The findings of these studies, therefore, may not necessarily be generalizable to those living outside of Western countries and cultures. In particular, Chinese society has not adequately perceived ACEs, such as exposure to domestic violence, as threats to child and youth development (20, 21). It is imperative to investigate whether ACEs affect Chinese as they did in Western samples. Given the lack of studies on ACEs and mindfulness in non-Western contexts, along with scholarship pointing to the importance of the college years to adult well-being (22–26), the primary aim of this study is to examine the effects of ACEs on mindfulness in a Chinese college student sample.

## Methods

### Data and Sample

The data for the present study came from an online anonymous survey administered to junior and senior students in 12 universities across China. We purposely selected universities from the north, east, south, west and middle regions of China to compile a representative sample. Once universities were selected, we contacted each university's department of social science and invited junior and senior students to participate in the online survey. A total of 2,229 students were invited for the survey in late September 2020. Reminders to participate in the survey were sent to students 3 and 7 days after the initial invitation. One thousand, eight hundred eighty-one students participated in the online survey by early October 2020. Ten surveys had incomplete answers and were excluded from the final analysis. Our final analytic sample contained data from 1,871 students, indicating a response rate of 80%. The research protocol was approved by the research review committee at one of the co-authors' University in China. An informed consent process was implemented prior to the survey. An incentive of 10 RMB for participation (2 USD) was provided. Students were informed that their participation was voluntary and that they could choose to stop completing the survey at any time.

### Measures

The dependent variable, *mindfulness*, was assessed by the 15-item Mindful Attention Awareness Scale (MAAS) (27). Past studies have shown that the Chinese version of MAAS is both valid and reliable for use with Chinese populations (9, 28). The 15 items asked participants to identify the frequency at which they experience feelings, behaviors, or mindful thoughts over the past 4 weeks. Examples of items include: “I rush through activities without being really attentive to them;” “I find myself doing things without paying attention;” and “I break or spill things because of carelessness, not paying attention, or thinking of something else.” The score for each item ranges from 1 to 6 (almost never to almost always). We reversed the scores so that higher scores indicated higher levels of mindfulness. The total of all scores provided ranged from 14 to 90, and the Cronbach's alpha of MAAS was 0.90 in this study.

The key independent variable, *adverse childhood experiences*, was measured by the Adverse Childhood Experience scale (ACE) and assessed adverse childhood experiences during the respondent's first 18 years of life (11). Ten items were used to measure ACEs across three dimensions: abuse (3 items), neglect (2 items), and household challenges (5 items). Example questions included “Did a parent or other adult in the household often: swear at you, insult you, put you down, or humiliate you?,” “Did you often feel that: No one in your family loved you or thought you were important or special?,” and “Did you live with anyone who was a problem drinker or alcoholic or who used street drugs?” Each affirmative answer was assigned one point. The questions were translated into Chinese by two Chinese doctoral students in the United States and verified by an American professor whose native language is Chinese. While the psychometrics of the Chinese language version of the ACE scale are not well-established, it has been used in previous studies (29, 30). The sum of all affirmative answers represents the ACE score. A higher score indicates a higher frequency of experiencing adverse events in the first 18 years of life. In addition, we calculate the scores of three dimensions in ACE scale, as well as the percentages of each and the total ACE events. The Cronbach's alpha of this scale was 0.69 in this study.

Students' demographic characteristics acted as controls in this study since previous studies have shown that they may affect ACEs and mindfulness (9, 16, 19, 30). These characteristics include age, gender (0 = male; 1 = female), ethnicity (1 = Han; 0 = other), household registration (rural, city with prior rural registration, city), parents' marital status (married, separated, divorced, and widowed), and highest educational background (elementary school or below, middle school, high school, and some college or above), number of family members, and annual family income and welfare status (0 = no; 1 = yes) in the last year. Given that the survey was conducted during the COVID-19 pandemic, we also controlled for COVID-19 experiences, which were measured by asking students whether their family members or friends had tested positive or died of COVID-19. Finally, since we sampled students from 12 colleges across China, different college characteristics may affect mindfulness of students as well. We thus take college into account by controlling for specific college characteristics, or college-fixed effect.

### Analytic Strategies

Descriptive analysis was performed to examine the distribution of each main variable. Next, to estimate the net effect of ACEs on mindfulness, we conducted multivariate regression analyses while controlling for socioeconomic characteristics of the students. The framework underlying this study posits that the degree of college students' mindfulness is determined by their ACEs as suggested by Herman's trauma theory (15), demographic characteristics, and college-level characteristics. The specification of the analytic model is represented by the following equation:

$$\begin{array}{l} Y_{\text{i}}=\alpha_{\text{i}}+\beta_{1}*\chi_{\text{i}}+C_{\text{i}}+\varepsilon_{\text{i}}, \end{array}$$

where *Y* i is mindfulness of the subject i; αi is the individual constant; χ is a vector of ACE and socioeconomic characteristics of subject i; Ci is the college for subject i, or college-fixed effect (which is taken to be constant across individual colleges); β is a vector of regression coefficients; and εi is the cross-section error component. Note that with college-fixed effect, the model controls for differences across colleges. Ordinary least squares (OLS) regression was used for the analyses. We conducted the regression analyses in several steps. First, to account for past literature indicating that both the occurrence of ACEs and number of ACEs may have different effects on mindfulness (16, 19), we examined how each specification may affect mindfulness in our sample. We compared the models' adjusted *R*-square values to determine which specification fit the data better and utilized the selected specification to conduct robustness tests with the ACE subscales and individual ACE items as independent variables. Given multiple regression analyses of different ACE specifications were performed, the Bonferroni test was used to control for multiple testing. The Bonferroni-adjusted *p*-value, calculated by multiplying the observed *p*-value by the number of tests performed, was used. STATA software 16.0 was used for all analyses.

## Results

### Descriptive Statistics

Table 1 presents the descriptive statistics of mindfulness and ACE. The sampled students had an average mindfulness score of 59.6. Scores ranged 15 to 90 and had a standard deviation of 10.8. About 35% of students (n=658) reported that they experienced at least one type of ACE in childhood. ACE scores in the sample ranged 0 to 10 with a mean of 0.69 (*SD* = 1.28). In our study, average ACE subscale scores were 0.28 (*SD* = 0.63) for abuse, 0.15 (*SD* = 0.41) for neglect, and 0.26 (*SD* = 0.61) for household challenges. With regards to individual ACE experiences, 14% of the sample reported parental separation or divorce. Other individual ACE experiences that the sample answered affirmatively at a high rate were emotional neglect (12%), emotional and sexual abuse (both at 11%), physical abuse (6%), and mental illness in the household (5%). The percentages of students reporting physical neglect, incarcerated household member, substance abuse in the household, and mother treated violently were low, all at 3% or below. Table 2 presents socioeconomic characteristics of the students. Less than 1% of students reported that they had family members or friends infected with COVID-19 (0.5%) or died (0.4%) of COVID-19. Due to low occurrence, we combined both infected and died into one category for regression analysis. Regarding the college composition, no college occupied the final sample more than 12%, ranged from 2.5 to 11.5%, reflecting the size of students in their social science departments.

**Table 1:** Level of mindfulness and adverse childhood experience.

	**Mean (S.D.) or *n* (%)**
Mindfulness (15–90)	59.61 (10.84)
Adverse Childhood Experience [*n*, %]	658, 35%
Adverse Childhood Experience [0–10]	0.69 (1.28)
Abuse [0–3]	0.28 (0.63)
Emotional abuse [*n*, %]	201, 11%
Physical abuse [*n*, %]	116, 6%
Sexual abuse [*n*, %]	213, 11%
Neglect [0–2]	0.15 (0.41)
Emotional neglect [*n*, %]	233, 12%
Physical neglect [*n*, %]	51, 3%
Household Challenge [0–5]	0.26 (0.61)
Parental separation or divorce [*n*, %]	264, 14%
Mother treated violently [*n*, %]	42, 2%
Substance abuse in the household [*n*, %]	37, 2%
Mental illness in the household [*n*, %]	88, 5%
Incarcerated household member [*n*, %]	51, 3%

*N = 1,871*.

**Table 2:** Descriptive statistics of socioeconomic variables.

	**Mean (S.D.) or *n* (%)**
**Gender [%]**	
Female	66.97
Male	33.03
Age	20.62 (0.96)
**Household registration [%]**	
Rural	38.70
City, rural before	8.93
City	52.37
**Grade [%]**	
Junior	60.72
Senior	39.28
**Ethnicity [%]**	
Han	89.36
Others	10.64
**Parent marital status [%]**	
Married	89.04
Separated	0.80
Divorced	6.89
Widowed	2.35
Others	0.91
**Parent highest education achievement [%]**	
Elementary school and below	6.90
Junior high school	28.11
High school	25.17
College and above	39.82
Family income	90,990 (122,030)
**Welfare status**	
No	74.72
Yes	25.28
Number of family members	3.87 (1.16)
**COVID-19 Infection in family and friends [%]**	
No	99.14
Infected	0.48
Dead	0.37
**College [%]**	
College 1	7.11
College 2	9.57
College 3	6.25
College 4	10.85
College 5	10.15
College 6	7.06
College 7	6.41
College 8	11.54
College 9	11.12
College 10	2.46
College 11	6.89
College 12	10.58

*N = 1,871*.

### Multivariate Analyses

Table 3 presents the standardized coefficients of mindfulness, estimated by OLS regression. Two models are presented. The first one modeled ACE as an occurrence variable [yes = 1, no = 0], while the second one used observed ACE score in the analysis. The occurrence of ACE had a significant and negative effect on mindfulness in Model 1. Students with ACE experience reported 0.16 standard deviation less mindfulness than students without ACE experience. In addition, age, parental marital status, and COVID-19 infection had significant effects, while HR and parental education had marginal effects, on mindfulness of students. Overall, level of mindfulness increased with age. Students whose parents were married had 0.05 standard deviation less mindfulness than their counterparts. Students who had family members or friends infected by COVID-19 also showed significantly lower mindfulness, by 0.09 standard deviation, than their counterparts. Mindfulness was also positively associated with city HR and students whose parents had college education or above. The adjusted *R*-square of Model 1 was 0.06. The adjusted *R*-square increased to 0.08 in Model 2. Model 2 showed that ACE score had a significant and negative effect on mindfulness. A one standard deviation increase in the ACE score was associated with a 0.21 standard deviation reduction in mindfulness. The rest of the results of Model 2 were similar to those reported in Model 1.

**Table 3:** Multivariate regression analysis of mindfulness.

	**Model 1**			**Model 2**		
	**Beta**	**S. E**.	***P***	**Beta**	**S. E**.	***P***
Adverse childhood experience [%]	−0.16	0.04	^***^	–	–	
Adverse childhood experience [Score]	–	–		−0.21	0.01	^***^
Female	0.00	0.04		0.00	0.04	
Age	0.06	0.02	^*^	0.06	0.02	^*^
Household registration: City, rural before	0.00	0.06		0.01	0.06	
Household registration: City	0.06	0.05	+	0.05	0.05	+
Junior	−0.01	0.04		−0.01	0.04	
Han	0.03	0.05		0.03	0.05	
Married	−0.05	0.06	^*^	−0.05	0.05	^*^
Junior high school	0.02	0.07		0.00	0.07	
High school	0.04	0.07		0.04	0.07	
College and above	0.10	0.08	+	0.09	0.08	+
Family income	−0.03	0.02		−0.04	0.02	
Welfare status	−0.01	0.04		−0.01	0.04	
Number of family members	−0.02	0.02		−0.02	0.02	
COVID-19 infection in family and friends	−0.09	0.18	^***^	−0.07	0.18	^**^
College fixed effects	Yes			Yes		
Adjusted *R*-square	0.06			0.08		

*N = 1,871. +p < 0.10*,

* *p < 0.05*,

** *p < 0.01*,

*** *p < 0.001*.

Given that Model 2 has a higher adjusted R-square, we conducted the same regression analyses for the three ACE subscales and individual ACE items. The robustness tests of ACE subscales and items on mindfulness are presented in Table 4. Each entry in Table 4 represents a different multivariate regression analysis with the same controls listed in Model 2 of Table 3. For simplicity, we only present the results for the ACE items in Table 4. The results for other variables were similar to those reported in Table 3. Each of the ACE subscales had a significant negative relationship with mindfulness, with the strongest effects from abuse (β = −0.17), followed by neglect (β = −0.16) and household challenge (β = −0.13). As for the individual ACE items, all items were associated with lower levels of mindfulness. Emotional abuse and neglect had the strongest effects on mindfulness (β = −0.16 for both), followed by physical and sexual abuse, and substance abuse in the household (β = −0.11 for all), physical neglect and mental illness in the household (β = −0.09), and mother treated violently (β = −0.07).

**Table 4:** Robustness tests of ACE subscales and items on mindfulness.

	**Mindfulness**			
	**Beta**	**S. E**.	***P***	**Adjusted *P***
**Whole ACE scale**				
Adverse childhood experience	−0.21	0.01	^***^	^***^
**Three dimensions**				
Abuse	−0.17	0.03	^***^	^***^
Neglect	−0.16	0.04	^***^	^***^
Household challenge	−0.13	0.03	^***^	^***^
**Individual items**				
Emotional abuse [0–1]	−0.16	0.05	^***^	^***^
Physical abuse [0–1]	−0.11	0.07	^***^	^***^
Sexual abuse [0–1]	−0.11	0.05	^***^	^***^
Emotional neglect [0–1]	−0.16	0.05	^***^	^***^
Physical neglect [0–1]	−0.09	0.10	^***^	^***^
Parental separation or divorce [0–1]	−0.07	0.06	^*^	
Mother treated violently [0–1]	−0.07	0.11	^**^	^*^
Substance abuse in the household [0–1]	−0.11	0.12	^***^	^***^
Mental illness in the household [0–1]	−0.09	0.08	^***^	^***^
Incarcerated household member [0–1]	−0.04	0.10	+	

*N = 1,871 +p < 0.10*,

* *p < 0.05*,

** *p < 0.01*,

*p <0.001*.

*Each entry in Table 4 represents a different multivariate regression analysis with the same controls listed in Model 2 of Table 3. For simplicity, we only present the results for the ACE items in Table 4. Adjusted P was the significance level of the observed p-value multiplied by the number of tests performed, 14*.

## Discussion

The findings of this study showed that the average mindfulness score (59.6) in our sample was lower than those of Chinese college students in previous studies (28, 31). Tan's et al. (31) sample of 508 Chinese college students had an average mindfulness score of 61.2 (*SD* = 11.1). Deng et al. (28) surveyed 263 students at Dalian University of Technology, China, and found an average mindfulness score of 63.6 (*SD* = 11.1). However, it is not clear whether the difference between our sample and others was due to COVID-19, timing, or sample composition. Future studies that use longitudinal research design to follow students over the course of the pandemic should be able to distinguish the changes of mindfulness before, during, and after the pandemic.

The mean ACE score in our sample was 0.69, which is lower than average ACE scores found in previous studies (29, 30). Notably, our sample and measures are not exactly comparable to previous studies. Zhang et al. (30) used rural high school graduates (*N* = 1,019) from 3 provinces in China and found that three-fourths of the sample reported at least one ACE. The sample averaged 1.6 ACEs (*SD* = 1.5). A systematic review of 32 studies on childhood maltreatment among Chinese college students found that 64.7% of students reported experiencing at least one form of childhood maltreatment (29). The extent to which low ACE prevalence in our sample was a result of sample differences or social desirability bias is unclear. Notably, our sample had a relatively higher socioeconomic background than other study samples. This warrants further investigation in future studies.

The findings from the regression analyses support trauma theory (15) and indicate modest effects of ACEs on students' mindfulness during the pandemic. Increasing the ACE score by one standard deviation was associated with a reduction of 0.21 standard deviations in mindfulness. In the ACE subscale analysis, all three subscales showed significant negative effects on mindfulness, with the strongest effect from abuse, followed by neglect and household challenges. In the ACE scale individual item analysis, emotional abuse, and neglect had the strongest effects on mindfulness, followed by physical and sexual abuse, and substance abuse in the household. The results indicate a modest influence of ACEs on the level of mindfulness for college students in China, particularly those students who report experiencing emotional abuse and neglect in the childhood.

Concerning the critical effects of ACEs on mindfulness, as well as other outcomes, researchers and practitioners should concentrate efforts into ACE prevention and protection as strategies to advance individuals' mindfulness and other life outcomes (18, 32, 33). In addition to the negative effect of ACEs on mindfulness found in this study, ACEs have been shown to be associated with negative outcomes, such as increased trauma, poor mental health, and greater delinquent and impulsive behaviors (12, 15, 33). To prevent ACEs, school social workers and practitioners can administer the ACE questionnaire annually to identify students with ACEs risk or at early stages of ACEs and provide services to them accordingly. In addition, as shown in our study, individuals who experienced neglect face similar harms to their mindfulness as those who experienced abuse. Despite neglect having been indicated as one of the most common forms of child maltreatment, professional, and scholarly attention has most often been directed to sexual and physical abuse, causing a “neglect of child neglect” (34). School social workers and practitioners need to pay attention to students with histories of neglect as they do to students who have been abused.

Mindfulness has been known to be an important protective factor for life development and various outcomes; recent studies have suggested that mindfulness may have a mediating effect on the relation between ACEs and these outcomes (4, 18, 32). Mindfulness, for example, is positively associated with self-regulation and emotional regulation, which may in turn allow individuals to better engage in the recognition, management, and resolution of overwhelming sensations, thoughts, and emotions. Mindful individuals can thus take the time to make decisions that promote their well-being. Indeed, past studies have reported the mediating effects of mindfulness on the association between ACEs and outcomes like alcohol use (32) and cyberbullying (18). Thus, the results of this study call for mindfulness-based interventions and services to students with high ACE scores as a strategy to buffer the negative effects of ACEs and to advance individuals' mindfulness and other life outcomes. Studies have shown that mindfulness-based stress reduction (MBSR), mindfulness-based cognitive therapy (MBCT), and mindfulness-based interventions (MBI) all can effectively reduce psychological distress and promote mental health and well-being (1, 35–38). For example, Joss et al. (37) adopted an 8 week MBSR program for young adults with a childhood maltreatment history and found that their changes in mindfulness positively correlated with their changes in self-compassion (*r* = 0.58, *p* = 0.001). Change in self-compassion were negatively correlated with changes in depression (*r* = −0.37, *p* = 0.05) and anxiety (*r* = −0.40, *p* < 0.05). The results support that the mindfulness-based intervention was helpful in improving self-compassion and psychological health of the sample (37). The majority of mindfulness-based interventions, however, focus on individuals who have experienced traumatic events and have psychopathological issues [e.g., (39–42)], while relatively fewer focus on the population of individuals who have experienced emotional abuse and neglect (43). The findings in this study underscore the importance of mindfulness-based interventions in potentially buffering the effects of ACEs on mindfulness and other life outcomes for those with experiences of child neglect.

With respect to the cultural and societal context of our study, it is notable that Chinese society has not sufficiently perceived ACEs, such as exposure to domestic violence, emotional abuse, and neglect, as threats to child and youth development yet (20, 21). Furthermore, influenced heavily by conservative and patriarchal family values, many may perceive ACEs as private family issues that should only be kept within the family and hidden from others (21, 44). Governments, agencies, and professionals should initiate various programs to promote public awareness of ACEs in China. For instance, community-based interventions could be a beneficial tool to advance public understanding and awareness of ACEs in communities and serve individuals who with a history of ACEs (45, 46). Home-based interventions should improve adults', particularly parents', knowledge of harmful family environments and positive parenting (47, 48).

This study has several limitations. First, our analyses were based on a cross-sectional dataset, which can only approximate an associative relationship, rather than a causal one, among ACE, COVID-19 infection, and mindfulness during the pandemic. Future research can use a longitudinal design to examine the causal relationship of these variables. Second, there were other unobserved variables, such as peer support and conflict, that could affect mindfulness but were not included in the study. The absence of these unobserved variables may have effects on the estimates reported in this study. Third, measures of mindfulness should comprehensively measure different dimensions of mindfulness. The measurement instrument used to assess mindfulness in this study, MAAS, was designed to measure the receptive state of mind (49). MAAS has been found to be positively related to emotional and behavioral regulation, and studies have found a high correlation between mindful attention and mindful metacognition (4, 49); however, some studies suggest that MAAS may lack construct validity (50–52). Future studies may therefore consider using a comprehensive and valid measure of mindfulness, such as Five Facet Mindfulness Questionnaire (FFMQ), which includes facets like non-judgmental inner experience and non-reactivity (53). Fourth, the findings of this study are based on data from social science departments in 12 colleges across China. Although the sample size and diversity of colleges across regions both increase our confidence, the extent to which these findings can be generalizable to all Chinese college students is unknown and requires further research. Finally, data gathered on key variables such as mindfulness and ACEs were from self-reports of the subjects. Self-reporting leaves our data subject to unintended and intended reporting errors, including social desirability bias, particularly for ACEs. Future studies might consider triangulating findings from different data sources, such as peer or teacher reports. Despite these limitations, the present study contributes to the knowledge on the factors that may contribute to mindfulness of Chinese college students during the COVID-19 pandemic.

## Conclusion

Empirical evidence has shown that mindfulness is associated with positive social and emotional competence, as well as academic performance in students (5–8). Meanwhile, the antecedents of mindfulness are relatively understudied (4, 8), though trauma theory (15) suggests that traumatic events, like ACEs, may very well-affect the mindfulness of individuals. This study analyzed data collected from 1,871 college students across China to investigate the extent to which ACEs affect mindfulness of college students. The findings of this study support the cross-cultural application of trauma theory (15) to a Chinese sample and indicate that ACEs significantly reduced students' mindfulness during the COVID-19 pandemic. The results underscore the importance of mindfulness-based interventions in potentially buffering the effects of ACEs on mindfulness, particularly for those with past experiences of emotional abuse and neglect and in a non-Western context such as China.

## Data Availability Statement

The raw data supporting the conclusions of this article will be made available by the authors, without undue reservation.

## Ethics Statement

The studies involving human participants were reviewed and approved by Rutgers University. The patients/participants provided their written informed consent to participate in this study.

## Author's Note

Mindfulness has been found to have many positive effects on life outcomes, including mental health and educational achievement. However, less is known about the antecedents of mindfulness, particularly in Chinese college students. This study examines the effect of adverse childhood experiences (ACEs) on mindfulness among Chinese college students in September 2020, during the COVID-19 pandemic. The data were collected from 1,871 college students from 12 colleges across China. The results indicate that ACEs and COVID-19 infection in family and friends were negatively associated with mindfulness. In particular, emotional abuse and neglect in childhood appear to have the most negative effects on mindfulness compared to other dimensions of ACEs such as physical abuse and household challenges. The findings of this article provide essential information to understand the antecedents of mindfulness in Chinese college students, especially during the COVID-19 pandemic, and offer vital implications for practice and recommendations for future research.

## Author Contributions

All authors listed have made a substantial, direct and intellectual contribution to the work, and approved it for publication.

## Conflict of Interest

The authors declare that the research was conducted in the absence of any commercial or financial relationships that could be construed as a potential conflict of interest.
